# Emergence of superconductivity in (NH_3_)_y_M_x_MoSe_2_ (M: Li, Na and K)

**DOI:** 10.1038/srep29292

**Published:** 2016-07-11

**Authors:** Xiao Miao, Saki Nishiyama, Lu Zheng, Hidenori Goto, Ritsuko Eguchi, Hiromi Ota, Takashi Kambe, Kensei Terashima, Takayoshi Yokoya, Huyen T. L. Nguyen, Tomoko Kagayama, Naohisa Hirao, Yasuo Ohishi, Hirofumi Ishii, Yen-Fa Liao, Yoshihiro Kubozono

**Affiliations:** 1 Research Laboratory for Surface Science, Okayama University, Okayama 700-8530, Japan; 2 Advanced Science Research Centre, Okayama University, Okayama 700-8530, Japan; 3 Department of Physics, Okayama University, Okayama 700-8530, Japan; 4 Research Centre of New Functional Materials for Energy Production, Storage and Transport, Okayama University, Okayama 700-8530, Japan; 5 Centre for Science and Technology under Extreme Conditions, Osaka University, Osaka 560-8531, Japan; 6 SPring-8/JASRI, Hyogo 679-5198, Japan; 7 National Synchrotron Radiation Research Center, Hsinchu 30076, Taiwan

## Abstract

We report syntheses of new superconducting metal-doped MoSe_2_ materials (M_x_MoSe_2_). The superconducting M_x_MoSe_2_ samples were prepared using a liquid NH_3_ technique, and can be represented as ‘(NH_3_)_y_M_x_MoSe_2_’. The *T*_c_s of these materials were approximately 5.0 K, independent of x and the specific metal atom. X-ray diffraction patterns of (NH_3_)_y_Na_x_MoSe_2_ were recorded using polycrystalline powders. An increase in lattice constant *c* showed that the Na atom was intercalated between MoSe_2_ layers. The x-independence of c was observed in (NH_3_)_y_Na_x_MoSe_2_, indicating the formation of a stoichiometric compound in the entire x range, which is consistent with the x-independence of *T*_c_. A metallic edge of the Fermi level was observed in the photoemission spectrum at 30 K, demonstrating its metallic character in the normal state. Doping of MoSe_2_ with Li and K also yielded superconductivity. Thus, MoSe_2_ is a promising material for designing new superconductors, as are other transition metal dichalcogenides.

Searching for new superconducting materials is one of the most challenging and exciting areas of research. During the past decade, iron pnictides (FeAs) and chalcogenides (FeSe) have attracted much attention, not only from researchers interested in developing new superconductors, but also physicists who are interested in the mechanism of superconductivity^1^,^2^,^3^,^4^. Recently, syntheses of metal-intercalated systems of FeSe using a liquid NH_3_ technique have been extensively studied because various superconductors with high superconducting transition temperatures (*T*_c_s) have been discovered^5^,^6^,^7^,^8^; the highest *T*_c_s are 46 K at ambient pressure^5^ and 49 K at high pressure^9^. The pressure-induced enhancement of *T*_c_ has also been confirmed for non-NH_3_ K_x_FeSe^10^. Thus a layered compound like FeSe is a promising material platform for investigating high-*T*_c_ superconductors.

The Mo dichalcogenide family has also attracted much attention because of the emergence of its unique physical properties^11^,^12^ and potential use in high-speed transistors^13^,^14^. Electrostatic electron-doping of MoS_2_ has produced superconductivity with a *T*_c_ as high as 10.8 K 11. The plot of *T*_c_ versus the accumulated two-dimensional (2D) electron density *n*_2D_ showed a dome-shaped curve, *i.e*., the *T*_c_ was tuned by the extent of electrostatic electron-doping. The maximum *T*_c_ was 10.8 K at 1.2 × 10^14^ cm^−2^. Also, a signature of 2D superconductivity was observed in electrostatically electron-accumulated MoS_2_^11^. The chemical doping of MoS_2_ with alkali and alkaline-earth metal atoms^15^,^16^ provided superconductivity with *T*_c_s lower than the maximum *T*_c_ of electrostatically electron-accumulated MoS_2_ 11,12. The chemical doping of MoS_2_ was achieved using the liquid NH_3_ technique, and many superconducting materials have been produced.

Very recently, electron-doping of MoSe_2_ was achieved by the electrostatic method^17^, and the *T*_c_ was precisely tuned in the same manner as in MoS_2_. In the case of MoSe_2_, only a Sr atom was intercalated, and MoSe_2_ then showed a *T*_c_ as high as 5.0 K^15^. This sample was prepared using the liquid NH_3_ technique, and the chemical composition of Sr_x_MoSe_2_ can be expressed as ‘(NH_3_)_y_Sr_x_MoSe_2_’, where the nominal x was 0.2. The shielding fraction of (NH_3_)_y_Sr_0.2_MoSe_2_ was 60%.

Here, we report syntheses of M_x_MoSe_2_ samples (M: Li, K and Na) using the liquid NH_3_ technique. In this study, Li, Na, K and Sr atoms were intercalated into MoSe_2_ solids (only Sr-intercalation had previously been reported)^15^. Single-crystal-like agglomerations of (NH_3_)_y_M_x_MoSe_2_ (M: Li, Na, K and Sr) were produced. Na-intercalation in (NH_3_)_y_Na_x_MoSe_2_ was indicated by its synchrotron powder X-ray diffraction (XRD) pattern. Energy dispersive X-ray spectroscopy (EDX) showed its chemical composition, and the amount of NH_3_ was also determined from the mass difference before and after reaction. The superconducting parameters were determined from the magnetic field (*H*) dependence of magnetization (*M*). The photoemission spectrum at 30 K showed a clear edge on the Fermi level, indicating metallic behavior in the normal state.

## Results

### Crystal structure of (NH_3_)_y_Na_x_MoSe_2_

Single crystals of pristine MoSe_2_ were prepared using the annealing technique; details are described in the Methods section. A photograph of a pure MoSe_2_ sample is shown in Figure S1a. A single-crystal structure analysis was produced using a piece of MoSe_2_ (or single crystal) separated from a MoSe_2_ agglomeration prepared in this study (Figure S1a); it is unclear whether an entire agglomeration is a single crystal or consists of multiple single crystals. A reasonable residual-factor (*R*) could be obtained in this analysis (*R* = 2.4% and weighted *R* (*wR*) = 4.6%). Only one phase of MoSe_2_ was included in the single crystal, and it was confirmed that no other phase such as Mo_3_Se_4_ was included. The structure of the MoSe_2_ single crystal was hexagonal (space group: No. 194, *P*6_3_/mmc). The lattice constants were *a* = 3.289(7) Å and *c* = 12.96(3) Å, which are consistent with those (*a* = 3.283 Å and *c* = 12.918 Å) reported previously for pristine MoSe_2_^18^. Crystallographic data are listed in Table S1. As seen from the magnetic susceptibility *M*/*H* (emu g^−1^ = cm^3^ g^−1^) shown in Figure S2, no superconductivity was observed in any precursor MoSe_2_ sample, implying no contamination with superconducting Mo_3_Se_4_. The chemical composition of one MoSe_2_ agglomeration was determined to be ‘MoSe_1.9(2)_’ from the EDX spectrum (Figure S3). These analyses also show that the precursor material was not superconducting Mo_3_Se_4_^19^, *i.e*., it was non-superconducting MoSe_2_. The EDX spectra, magnetic susceptibilities and single-crystal analyses guaranteed that all MoSe_2_ agglomerations used for metal-intercalation throughout this study were in fact substantially ‘MoSe_2_’.

Metal-doped MoSe_2_ samples were prepared using the liquid NH_3_ technique. The experimental details are described in the Methods section. Here, it is worth noting that instead of a polycrystalline powder, in this study, an agglomeration of MoSe_2_ was used as the starting material for metal-intercalation. This is based on the successful synthesis of metal-doped FeSe from an agglomeration of FeSe^20^.

**Figure 1:**
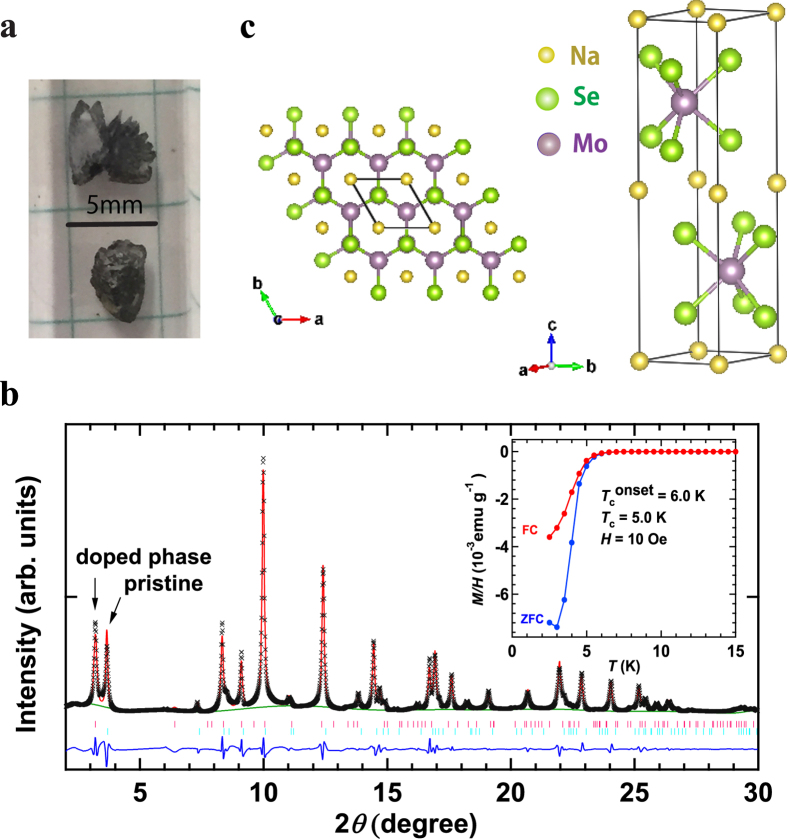
(**a**) Photograph of (NH_3_)_y_Na_0.5_MoSe_2_ agglomerations. (**b**) Powder XRD pattern of (NH_3_)_y_Na_0.5_MoSe_2_ using synchrotron radiation. ‘x’ marks correspond to the experimental XRD pattern. Red and green lines refer to calculated patterns (Le Bail fitting) and background, respectively. Ticks refer to the peak positions predicted. In (**b**), two phases ((NH_3_)_y_Na_x_MoSe_2_ and MoSe_2_) are used in Le Bail fitting. The *M*/*H* – *T* plots in ZFC and FC modes for the (NH_3_)_y_Na_0.5_MoSe_2_ sample providing the XRD pattern (**b**) are shown in the inset of (**b**). (**c**) Schematic representation of possible (NH_3_)_y_Na_0.5_MoSe_2_ structure; the structure was drawn based on the atomic coordinates shown in Table S2. As described in the text, this structure may be reasonable if the Na is located in the space between MoSe_2_ layers, a possibility supported by the expansion of (**c**).

A photograph of (NH_3_)_y_Na_0.5_MoSe_2_ prepared using the liquid NH_3_ method is shown in Fig. 1a; the stoichiometry of Na (x = 0.5) is an experimental nominal value. The (NH_3_)_y_Na_0.5_MoSe_2_ samples (agglomerations) look like single crystals. The EDX spectrum for (NH_3_)_y_Na_0.5_MoSe_2_ is shown in Figure S4, which shows that the (NH_3_)_y_Na_0.5_MoSe_2_ sample is (NH_3_)_0.4(1)_Na_0.41(1)_MoSe_2.04(1)_. The amount of NH_3_, y = 0.4(1), was determined from the mass difference before and after the reaction that used liquid NH_3_. These results indicate that NH_3_ was included in this material, and the amount of Na is reasonably consistent with the experimental nominal value. Here, we must consider the exact chemical structure and appropriate representation of NH_3_, *i.e*., which form exists in the MoSe_2_ solid? Does it exist as molecular NH_3_, or does it take some other form such as a metal-coordinated amide? To determine the exact chemical formula, neutron diffraction may be required. Throughout this paper the simple chemical formula, (NH_3_)_y_M_x_MoSe_2_, is used for convenience because the exact chemical form of NH_3_ is unclear.

The structure of (NH_3_)_y_Na_0.5_MoSe_2_ (0.5 is a nominal experimental value) was examined as a typical example using single-crystal XRD data collected at room temperature. As seen in Figure S1b, the XRD Bragg spots are quite diffuse, indicating a very disordered crystal. Because of the diffuse spots, a definitive structural analysis could not be performed.

To confirm whether the Na atom is located midway in the space between MoSe_2_ layers, the powder XRD pattern of (NH_3_)_y_Na_0.5_MoSe_2_ was measured with synchrotron radiation (λ = 0.4137(1) Å). The XRD pattern is shown in Fig. 1b together with the pattern calculated based on Le Bail fitting. The Le Bail fitting was performed for two phases under the space group of *P*6_3_/mmc. The sample was prepared from Na and MoSe_2_ using the liquid NH_3_ technique, and ground up for the acquisition of a powder XRD pattern. The *a* and *c* of the main phase were determined to be 3.541(2) and 14.810(4) Å, respectively, while those of the minor phase were 3.2615(1) and 12.8133(5) Å. The minor phase can be assigned to pure MoSe_2_, the lattice constants of which are consistent with the values (*a* = 3.289(7) Å and *c* = 12.96(3) Å) determined for pure MoSe_2_ single crystal in this study. As seen from Fig. 1b, the peak-intensity of 002 peaks for non-doped (minor) and Na-doped MoSe_2_ (major) observed at angles below 2*θ* = 5° were virtually the same, indicating that the fractions were almost equivalent. No other phase (such as metal-doped Mo_3_Se_4_) was found, which is reasonable because the precursor material before metal-doping was demonstrated to be MoSe_2_.

The *c* of 14.810 Å of the main phase is larger by 1.85 Å than that of pure MoSe_2_ (12.96(3) Å), indicating that the Na is located in the space between MoSe_2_ layers. The *a* value also increased to 3.541(2) Å from 3.289(7) Å, but the expansion (Δ*a* = 0.252 Å) is too small to be attributed to the intercalation of Na into the MoSe_2_ layer. As discussed later, the intercalation of Na at a 2*a* site, *i.e*., the space between MoSe_2_ layers, seems to be the most reasonable explanation of the observed changes. The *R* and weighted pattern *R* (*wR*_p_) were 3.2 and 4.8% in the Le Bail fitting, respectively, which are reasonable values that confirm the Le Bail analysis. The structure suggested is shown in Fig. 1c; in this structure, NH_3_ is not shown. A more precise crystal structure that includes NH_3_ must be determined using high-quality (NH_3_)_y_Na_0.5_MoSe_2_ single crystals that yield sharp Bragg spots. This study is now in progress.

In this study, we tried to perform Rietveld refinement based on the model listed in Table S2 of Supplementary Information; the atomic coordinates listed in Table S2 were obtained by a structural analysis based on single-crystal X-ray data, but a reasonable *R* factor could not be obtained in the analysis because of the diffuse Bragg spots collected from the single crystal (Figure S1b). The complete Rietveld refinement could not be achieved using the above model, so it was not possible to determine the exact location of the Na atom. However, the large expansion of *c* suggests that Na is located in the space between MoSe_2_ layers. If this is the case, the location of Na at a 2*a* site may be reasonable because of the presence of a large space around the 2*a* site. A possible crystal structure of (NH_3_)_y_Na_x_MoSe_2_ is shown in Fig. 1c.

### Characterization of superconductivity in (NH_3_)_y_Na_x_MoSe_2_

**Figure 2:**
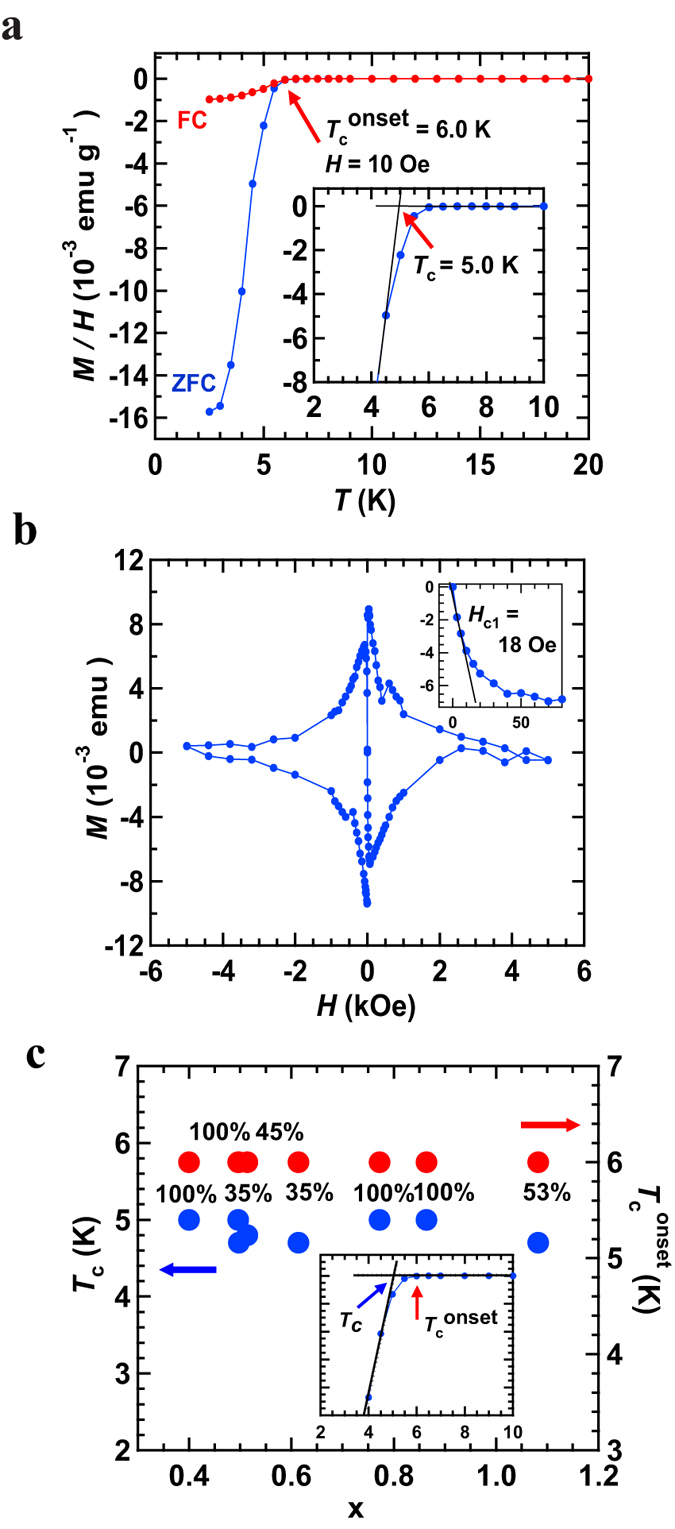
(**a**) *M*/*H vs*. *T* plots of the (NH_3_)_y_Na_0.5_MoSe_2_ agglomerations in ZFC and FC modes (*H* = 10 Oe). Inset in (**a**) shows the method used to determine *T*_c_. (**b**) *M* – *H* curve measured at 2 K for the (NH_3_)_y_Na_0.5_MoSe_2_ agglomerations. In the inset of (**b**), the expanded *M* – *H* curve is shown together with the fitted line. The chemical composition of (NH_3_)_y_Na_0.5_MoSe_2_ used in (**a**,**b**) was determined to be (NH_3_)_0.4(1)_Na_0.41(1)_MoSe_2.04(1)_ (see text). (**c**) x-dependence of *T*_c_ and *T*_c_^onset^ in (NH_3_)_y_Na_x_MoSe_2_; x was evaluated from the EDX. In (c) the shielding fraction is evaluated using the ρ determined using each chemical stoichiometry for (NH_3_)_y_Na_x_MoSe_2_; y is assumed to be 0.4. The inset of (**c**) shows how to determine the *T*_c_ and *T*_c_^onset^.

Figure 2a shows the *M*/*H* – temperature (*T*) curves in zero field cooling (ZFC) and field-cooling (FC) modes for (NH_3_)_0.4(1)_Na_0.41(1)_MoSe_2.04(1)_. The *T*_c_^onset^ and *T*_c_ were 6.0 and 5.0 K, respectively, for (NH_3_)_0.4(1)_Na_0.41(1)_MoSe_2.04(1)_; the *T*_c_ was determined from the crossing point of the extrapolation of the normal state and the drop of the *M*/*H* – *T* curve in ZFC mode, as seen from the inset in Fig. 2a. Here, it may be necessary to briefly comment on a small slow decrease in *M*/*H* below *T*_c_^onset^ (Fig. 2a). The inhomogeneous Na-doping of MoSe_2_ may be suggested as its origin. However, as described later, the different x values in (NH_3_)_y_Na_x_MoSe_2_ did not provide different *T*_c_ or *T*_c_^onset^ values, which means that the inhomogeneous Na-doping cannot explain the slow decrease. The second possibility is that the (NH_3_)_y_Na_x_MoSe_2_ agglomerations shown in Fig. 1a are not single crystals but aggregates of polycrystalline grains because the small size of superconducting grains often results in such a decrease. These possibilities are fully explored later.

The shielding fraction at 2.5 K was 100% for (NH_3_)_0.4(1)_Na_0.41(1)_MoSe_2.04(1)_; the shielding fraction was evaluated using the density (*ρ* = 5.64 g cm^−3^) determined from the above chemical stoichiometry and lattice constants shown in the previous section. Here it should be noted that the above sample was made by Na-doping of an agglomeration of MoSe_2_. As a reference, the *M*/*H* – *T* plot of the (NH_3_)_y_Na_0.5_MoSe_2_ sample prepared by Na-doping of polycrystalline MoSe_2_ powder is shown in Figure S5 of Supplementary Information. The *T*_c_ and *T*_c_^onset^ (Figure S5) were the same as those (Fig. 2a) of a sample prepared by Na-doping of a MoSe_2_ agglomeration, but the shielding fraction was less than 1% at 2.5 K. The behavior of the *M*/*H* – *T* plot below *T*_c_^onset^ (Figure S5) was also the same as that shown in Fig. 2a. These results may show that effective Na-doping can be performed on these agglomerations of MoSe_2_. Moreover, we suggest that the above small fraction (<1%) may originate in a limiting thickness of superconductivity, *i.e*., a thin superconducting area formed by metal-doping using polycrystalline MoSe_2_ powder. Therefore, throughout this paper, all studies were performed using the samples prepared by metal-doping of agglomerations of MoSe_2_.

Finally, we comment briefly on the Meissner fraction of (NH_3_)_0.4(1)_Na_0.41(1)_MoSe_2.04(1)_ at 2.5 K (shielding fraction = 100% at 2.5 K (Fig. 2a)). The Meissner fraction was approximately 6.7% at 2.5 K which was evaluated from the *M*/*H* – *T* plot in FC mode (Fig. 2a), indicating a small size for superconducting grains. Therefore, this single-crystal like (NH_3_)_0.4(1)_Na_0.41(1)_MoSe_2.04(1)_ may actually consist of polycrystalline superconducting grains, as previously suggested based on the slow drop observed in the *M*/*H* – *T* plot below *T*_c_^onset^ (Fig. 2a). However, some of (NH_3_)_y_Na_x_MoSe_2_ samples showed a Meissner fraction of more than 20%. Figure S6 shows *M*/*H* – *T* plots of (NH_3_)_y_Na_0.5_MoSe_2_ exhibiting a Meissner fraction of 25%.

Figure 2b shows the *M* – *H* curve at 2 K for (NH_3_)_0.4(1)_Na_0.41(1)_MoSe_2.04(1)_, which exhibits a clear diamond-like shape. The lower critical field *H*_c1_ was determined to be 18 Oe from the expanded *M* – *H* curve (inset of Fig. 2b). It was concluded from the *M* – *H* curve (Fig. 2b) that the upper critical field, *H*_c2_, was > 0.3 T, indicating a type-II superconductor. Figure S7 shows *M*/*H* – *T* plots at different *H*’s, and the *H* – *T* phase diagram (Figure S7) was constructed from the *T*_c_^onset^ at each *H*; the fitted curve indicates the *H*_c2_ at each temperature. The positive curvature seen in Figure S7 is similar to the behavior of (NH_3_)_y_K_x_MoS_2_ reported recently^21^. The *H*_c2_ at 0 K, *H*_c2_(0), was evaluated to be 2.4 T. However, the data of the *H*_c2_ – *T*_c_ plot are confined near *T*_c_. Therefore, the *H*_c2_ is shown just for reference. We determined the London penetration depth, λ, to be 520 nm, from *H*_c1_. The shape of the sample was assumed to be isotropic because the measurements of *M* – *H* (2 K) and *M*/*H* – *T* at different *H*’s was performed using more than one agglomerations.

Figure 2c shows the x dependence of *T*_c_ in (NH_3_)_y_Na_x_MoSe_2_. The x value was determined from the EDX spectrum, and the x refers to the statistically averaged value with a small error bar falling within the range of the circle (Fig. 2c); the EDX was measured for several areas in one sample. The *T*_c_ was almost constant (~5 K) with an x-range of 0.4–1. The shielding fraction was higher than 35% in all samples. For the discussion, we plotted *T*_c_^onset^ − x in Fig. 2c again because the previous reports on metal-doped MoS_2_ and MoSe_2_ show the *T*_c_^onset^. The *T*_c_^onset^ was also constant (~6 K) in the x-range of 0.4–1. Therefore, we cannot point to an x-dependence of superconductivity in (NH_3_)_y_Na_x_MoSe_2_. Finally, we must comment that the maximum x is 1.0 in (NH_3_)_y_Na_x_MoSe_2_ if the Na occupies only a 2*a* site in the *P*6_3_/mmc lattice, as described in the subsequent section. To sum up, it must be stressed that the x range must be 0–1 in (NH_3_)_y_Na_x_MoSe_2_. A list of typical superconducting samples is shown in Table 1.

### Electronic structure of (NH_3_)_y_Na_x_MoSe_2_

**Figure 3:**
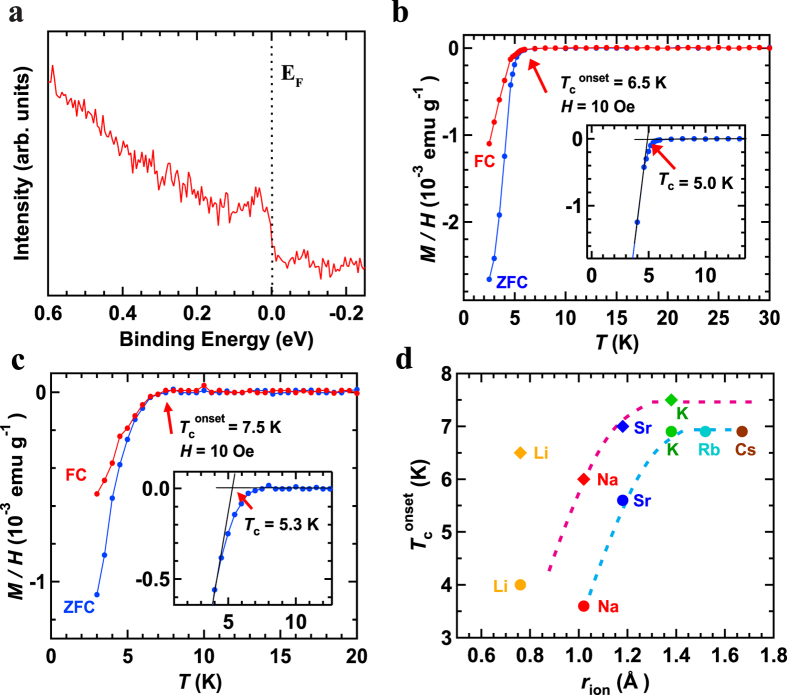
(**a**) Photoemission spectrum of (NH_3_)_y_Na_0.5_MoSe_2_. *M*/*H* versus *T* plots of (**b**) (NH_3_)_y_Li_0.5_MoSe_2_ and (**c**) (NH_3_)_y_K_0.5_MoSe_2_ agglomerations, respectively, in ZFC and FC modes (*H* = 10 Oe). (**d**) Plot of *T*_c_^onset^ *vs*. *r*_ion_ in (NH_3_)_y_M_x_MoSe_2_ and (NH_3_)_y_M_x_MoS_2_. Circles and diamonds refer to (NH_3_)_y_M_x_MoS_2_ and (NH_3_)_y_M_x_MoSe_2_, respectively. The plot is based on the data collected in this study (diamonds) and those in Refs 15 and 16 (circles).

The photoemission spectrum of a single-crystal-like agglomeration of (NH_3_)_y_Na_0.5_MoSe_2_ measured at 30 K is shown in Fig. 3a; the spectrum was recorded at the Γ point using the Xe-Iα resonance line (8.44 eV). The photoemission intensity was observed on the Fermi level, *i.e*., the metallic edge was clearly recorded. This shows that (NH_3_)_y_Na_0.5_MoSe_2_ is metallic in the normal state, and the superconducting transition of (NH_3_)_y_Na_0.5_MoSe_2_ emerges from the metallic state. The evaluation of the superconducting gap in (NH_3_)_y_Na_0.5_MoSe_2_ has not yet been done due to the limited resolution of 15 meV in the photoelectron spectrometer, so this is future work. While the metallic edge was clearly observed in the normal state by Xe-Iα light, no signature of the metallic edge was obtained when changing Xe-Iα to the He-Iα resonance line (21.2 eV). We note that the surface of the (NH_3_)_y_Na_x_MoSe_2_ single crystal may be oxidized, as the photoemission spectrum using the Xe-Iα resonance line provides more bulk-sensitive results than He-Iα. The successful observation of the metallic edge at the Γ point is fully treated in the Discussion section.

### Superconductivity in other metal-intercalated MoSe_2_

Figure 3b,c show the *M*/*H* – *T* curves for (NH_3_)_y_Li_0.5_MoSe_2_ and (NH_3_)_y_K_0.5_MoSe_2_, in ZFC and FC modes. The *T*_c_^onset^ and *T*_c_ were 6.5 and 5.0 K, respectively, for (NH_3_)_y_Li_0.5_MoSe_2_, and were 7.5 and 5.3 K for (NH_3_)_y_K_0.5_MoSe_2_. The shielding fraction at 2.5 K was 21% for (NH_3_)_y_Li_0.5_MoSe_2_, and 10.5% for (NH_3_)_y_K_0.5_MoSe_2_. These shielding fractions were roughly estimated using the *ρ* ( = 6.99 g cm^−3^) of MoSe_2_ because the exact *ρ* could not be determined for (NH_3_)_y_Li_0.5_MoSe_2_ and (NH_3_)_y_K_0.5_MoSe owing to the absence of structural data (lattice constants). Therefore, the values may be slightly overestimated, but the shielding fraction suggests that the superconducting phases can be formed by intercalating alkali metal atoms other than Na. The *T*_c_^onset^s of these materials were higher than the 6 K of (NH_3_)_y_Na_0.5_MoSe_2_. However, the *T*_c_ was almost the same for three (NH_3_)_y_M_x_MoSe_2_’s. Furthermore, we synthesized the superconducting (NH_3_)_y_Sr_x_MoSe_2_ (nominal x = 0.2), which showed a *T*_c_ (*T*_c_^onset^) as high as 4.8 K (7.0 K) (*M*/*H* – *T* plots not shown); the *T*_c_ was the same as that reported previously^15^. The shielding fraction was ~2.5% at 2.5 K which is lower than those of alkali-metal-doped MoSe_2_.

In the case of (NH_3_)_y_M_x_MoS_2_, the *T*_c_^onset^ generally increases with an increase in *c*^15^, and it increases with the ionic radius (*r*_ion_) of the intercalant. However, the *T*_c_^onset^ of (NH_3_)_y_Li_x_MoS_2_ deviates from this pattern^15^. The *T*_c_^onset^ vs. *r*_ion_ for (NH_3_)_y_M_x_MoSe_2_ (M: Li, Na, Sr and K) is plotted in Fig. 3d, together with that of (NH_3_)_y_M_x_MoS_2_ reported previously^15^,^16^. Similar behavior is seen in the plots of *T*_c_^onset^ − *r*_ion_ of (NH_3_)_y_M_x_MoSe_2_ and (NH_3_)_y_M_x_MoS_2_. The *T*_c_^onset^ of (NH_3_)_y_Li_x_MoSe_2_ deviates from the suggested relationship, as does that of (NH_3_)_y_Li_x_MoS_2_ 15. We briefly tried to synthesize (NH_3_)_y_M_x_MoSe_2_ (M: Rb, Cs, Ca, Ba, Sr and Yb) as well as (NH_3_)_y_Li_0.5_MoSe_2_, (NH_3_)_y_Na_0.5_MoSe_2_ and (NH_3_)_y_K_0.5_MoSe_2_. At the present stage, their superconductivity has not yet been observed, except for (NH_3_)_y_Sr_x_MoSe_2_ which was previously reported^15^.

## Discussion

Very recently, Shi *et al*. succeeded in achieving superconductivity through electrostatic electron-doping of MoSe_2_^17^. The maximum *T*_c_ of MoSe_2_ reaches 7.1 K at *n*_2D_ = 1.69 × 10^14^ cm^−2^, and the *T*_c_ can be tuned by the accumulated electron density. The maximum *T*_c_ is lower than the 10.8 K of MoS_2_^11^ and the *n*_2D_ is higher than the 1.2 × 10^14 ^cm^−2^ of MoS_2_^11^. For MoSe_2_, a dome-like phase diagram of *T*_c_ vs. *n*_2D_ has not yet been observed because the number of metal-doped MoSe_2_ superconductors discovered is still small, *i.e*., a *T*_c_ in the *n*_2D_-range (>1.69 × 10^14^ cm^−2^), which will be achieved by chemical electron-doping, has not yet been plotted.

**Figure 4:**
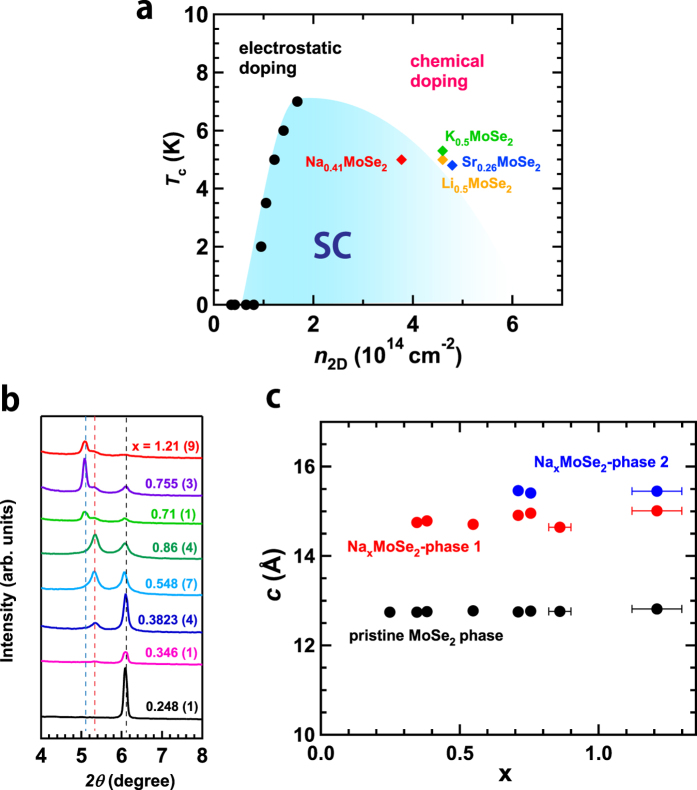
(**a**) Phase diagram of electron-accumulated MoSe_2_. This phase diagram is based on the *T*_c_^onset^ (diamonds) of (NH_3_)_y_M_x_MoSe_2_ (this work) and those (circles) of electrostatically electron-accumulated MoSe_2_ recently reported by Shi *et al*.^17^ ‘(NH_3_)_y_’ is omitted in the formulas identifying differently M-intercalated (NH_3_)_y_M_x_MoSe_2_. (**b**) XRD patterns of (NH_3_)_y_Na_x_MoSe_2_ samples with different x; each x was determined from the EDX spectrum. The peaks at 2*θ* = 6.1°, 5.4° and 5.1° correspond to *002* peaks due to non-doped MoSe_2_, (NH_3_)_y_Na_x_MoSe_2_ and another (NH_3_)_y_Na_x_MoSe_2_ phases, respectively. (**c**) x-dependence of *c* for the above three phases. The *c* values do not change with x.

A fresh *T*_c_ − *n*_2D_ diagram (Fig. 4a) was prepared using the *T*_c_ − *n*_2D_ plot (electrostatic electron-doping) reported by Shi *et al*.^17^ and the *T*_c_ − *n*_2D_ plot (chemical electron-doping) for (NH_3_)_y_M_x_MoSe_2_ samples produced in this study. Here, it should be noted that the 3D electron density, *n*_3D_, evaluated from the x and lattice volume in (NH_3_)_y_Na_x_MoSe_2_ was translated to 2D electron density *n*_2D_ by assuming the thickness of the channel region to be one layer ( = *c*/2); the electron concentration donated from a metal atom to the MoSe_2_ layer was evaluated assuming that an alkali (alkali-earth) metal atom can donate only one (two) electron, *i.e*., complex processes such as back-electron transfer to NH_3_ were not considered. This is the same method used for the estimation of the *T*_c_ − *n*_2D_ plot for metal-doped MoS_2_ 17. In the phase diagram, the *T*_c_s of (NH_3_)_y_Li_0.5_MoSe_2_, (NH_3_)_y_K_0.5_MoSe_2_ and (NH_3_)_y_Sr_0.261(1)_MoSe_2_ are also plotted for reference, although the x is an experimental nominal value except in (NH_3_)_y_Sr_0.261(1)_MoSe_2_. Consequently, a dome-like phase diagram was suggested in the same manner as MoS_2_^11^, but a continuous change of *T*_c_ was not obtained in the high *n*_2D_ range because of the almost identical *T*_c_ in metal-doped MoSe_2_ prepared in this study (Fig. 4a).

As described in the Results section, the *T*_c_^onset^ increases with increasing *r*_ion_ (Fig. 3d). This behavior is contrary to that of (NH_3_)_y_M_x_FeSe, in which the *T*_c_^onset^ is inversely proportional to the *r*_ion_^7^. In the case of (NH_3_)_y_M_x_FeSe, the *T*_c_ is closely associated with the FeSe plane spacing ( = *c*/2)^7^,^8^,^9^, and elements with a smaller *r*_ion_ produced larger FeSe plane spacings. This strange behavior can be explained by the fact that the crystal structure differs (off-center or on-center structures) depending on the *r*_ion_ of the intercalated element^8^, so that (NH_3_)_y_Li_x_FeSe, with an off-center structure, provides a larger FeSe plane spacing and high *T*_c_ (~44 K)^5^,^8^. If the *T*_c_ (or *T*_c_^onset^) also depends on the MoSe_2_ plane spacing in (NH_3_)_y_M_x_MoSe_2_, the graph shown in Fig. 3d implies that an increase in the *r*_ion_ of the intercalant directly affects the MoSe_2_ plane spacing. Actually, the deviation of *T*_c_^onset^ of (NH_3_)_y_Li_0.5_MoSe_2_ and (NH_3_)_y_Li_0.5_MoS_2_ from the *T*_c_^onset^ − *r*_ion_ curve drawn in the graph shown in Fig. 3d may imply that (NH_3_)_y_Li_x_MoSe_2_ adopts a different structure from that (see Fig. 2c) determined for (NH_3_)_y_Na_x_MoSe_2_. In other words, we expect a different location for the Li atom in (NH_3_)_y_Li_x_MoSe_2_ than that of the Na atom (probably 2*a* site), as found in (NH_3_)_y_Li_x_FeSe^6^,^8^. To sum up, we must discuss the superconductivity of (NH_3_)_y_M_x_MoSe_2_ in the light of two variables, *n*_2D_ and MoSe_2_ plane spacing (or two dimensionality). This makes it difficult to observe a dome-like *T*_c_ − *n*_2D_ phase diagram, as seen from Fig. 4a.

As described in the Results section (Fig. 2c), no x-dependence of *T*_c_ (or *T*_c_^onset^) was observed in (NH_3_)_y_Na_x_MoSe_2_. Here, it is very interesting and significant to investigate whether the lattice constants (*a*,*c*) change with the x value in (NH_3_)_y_Na_x_MoSe_2_. Figure 4b shows the expanded X-ray diffraction patterns (2*θ* = 4.0–8.0°), indicating that the 002 peaks due to doped and non-doped phases are observed at the constant 2*θ* values although the peak intensity due to the doped phase increases monotonically with increasing x in the x-range of 0.35 to 0.86. From this result, it was found that the *c* does not change with x, suggesting that the stoichiometric (NH_3_)_y_Na_x_MoSe_2_ is formed regardless of any increase in x. In other words, the chemical stoichiometry of (NH_3_)_y_Na_x_MoSe_2_ does not change even when x increases, and only the fraction of the non-doped phase decreases. Such behavior was recently observed in (NH_3_)_y_K_x_MoS_2_ 21, in which the K_0.4_MoS_2_ (2H structure) and K_1.0_MoS_2_ (1T and 1T’ structure) are formed in low and high K concentrations, respectively. The constant *T*_c_ may be reasonably explained by the scenario that the stoichiometric (NH_3_)_y_Na_x_MoSe_2_ compound (or the chemical compound with fixed x and y) is formed in the entire x range, *i.e*., the stoichiometric x value in (NH_3_)_y_Na_x_MoSe_2_ does not change with increasing x as determined from EDX; the EDX estimates the x value including non-intercalated Na atoms. This scenario corresponds to the third possibility described in the Results section.

As seen from Fig. 4b, at higher x values than 0.7, a new peak was observed, indicating the presence of a new *c*-expanded phase. Figure 4c shows the x-dependence of *c* in (NH_3_)_y_Na_x_MoSe_2_. From this graph, three different *c* values are found, due to (1) non-doped pure MoSe_2_, (2) a Na-doped MoSe_2_ phase, and (3) another Na-doped MoSe_2_ phase with a larger MoSe_2_ spacing. Since the *T*_c_ did not change in the entire x-range regardless of the formation of phase (3), it was unclear whether phase (3) is a new superconducting phase. To sum up, when x increases, two different Na-doped MoSe_2_ phases with certain chemical stoichiometry seem to be formed in (NH_3_)_y_Na_x_MoSe_2_. Further study is necessary to clarify the exact stoichiometry of their phases.

Finally, it is necessary to comment on the observation of a metallic edge on the Fermi level in the photoelectron spectrum measured at the Γ point. The band dispersion in bulk crystals of pure MoSe_2_ shows an indirect band gap (Γ – (ΓK))^22^, where (ΓK) means an intermediate state between Γ and K. However, the band dispersion in a single layer of MoSe_2_ shows a direct band gap (K – K)^22^. Therefore, a metallic edge for (NH_3_)_y_Na_x_MoSe_2_ should be observed at the (ΓK) point for MoSe_2_ crystal if we assume a rigid-band picture of band dispersion. Furthermore, even if we assume a single-layer like MoSe_2_ accompanied by expansion of the spacing between MoSe_2_ layers due to Na-intercalation, a metallic edge must be observed at the K point. Therefore, a metallic edge should not be observed at the Γ point. Nevertheless, a metallic edge was clearly observed in the photoemission spectrum (Fig. 3a). Relevant to this question, it can be observed that the photoemission spectrum must detect all band dispersion of (NH_3_)_y_Na_0.5_MoSe_2_ since the single crystal of MoSe_2_ must be disordered to possess different crystal alignments. In other words, the photoemission spectrum of a polycrystalline-like (NH_3_)_y_M_x_MoSe_2_ granule is recorded in Fig. 3a. This interpretation is reasonable since some disorder in the crystal is suggested by the XRD pattern shown in Figure S1b.

## Methods

### Sample preparation and characterization

Single crystals of MoSe_2_ were formed from a polycrystalline powder MoSe_2_ sample by physical vapor transport using a furnace with different temperature zones^23^; the powder MoSe_2_ sample was prepared by annealing stoichiometric amounts of Mo and Se at 800 °C for 3 days and 1000 °C for 4 days, according to a procedure reported elsewhere^23^. To form single crystals of MoSe_2_, TeCl_4_ was mixed with a MoSe_2_ sample as a transport material, the powder MoSe_2_ sample was set in the 1000 °C source area, and MoSe_2_ single crystals were collected in the low-temperature zone at 900 °C. Here we have used the term ‘MoSe_2_ single crystal’, but actually it is unclear whether the entirety of an agglomeration consists of one single crystal. Therefore, instead of the term ‘single crystal’, it may be valid to use the term ‘agglomeration of MoSe_2_’.

The samples of (NH_3_)_y_M_x_MoSe_2_ (M: Na, Li and K) were synthesized by the liquid NH_3_ technique as follows: (1) stoichiometric amounts of MoSe_2_ agglomerations and an alkali metal were placed in a glass tube, and then NH_3_ gas was condensed in the tube. (2) The metal dissolved in the liquid NH_3_ at −60 °C, and the solution (colored blue) was kept below −50 °C for 6 days. (3) When the color disappeared, the NH_3_ was removed by dynamical pumping at room temperature. The same method was used for Sr-intercalation in MoSe_2_.

The DC magnetic susceptibility (*M*/*H*) of all samples was measured using a SQUID magnetometer (Quantum Design MPMS2). The single-crystal XRD patterns of the samples were measured with a Rigaku Saturn 724 diffractometer with a Mo *K*α source (wavelength λ = 0.71078 Å). The powder XRD patterns of (NH_3_)_y_Na_0.5_MoSe_2_ and (NH_3_)_y_Na_x_MoSe_2_ (x = 0–1) were obtained using synchrotron radiation (λ = 0.4137(1) Å) from the BL10XU beamline and (λ = 0.6887 Å) from the BL12B2 beamline, respectively, of the Spring-8 in Japan; the incident beam was focused by a stacked compound X-ray refractive lens. The samples were introduced into quartz tubes in an Ar-filled glove box for *M*/*H* measurements, or into capillaries for XRD; the quartz tubes were pumped and sealed under vacuum, while the capillaries were sealed under Ar atmosphere. The EDX was obtained with an EDX spectrometer equipped with a scanning electron microscope (SEM) (KEYENCE VE-9800 - EDAX Genesis XM_2_), and the photoemission spectrum with a SCIENTAOMICRON R4000 analyzer and a discharge lamp (SPECS). The Fermi level of the sample was referenced to that of gold, which was in electrical contact with the sample. The sample was cleaved in the ultrahigh-vacuum chamber for the measurement of photoemission spectrum. The photoemission spectrum was measured in an ultrahigh vacuum of ~5 × 10^−9^ Pa.

## Additional Information

**How to cite this article**: Miao, X. *et al*. Emergence of superconductivity in (NH_3_)_y_M_x_MoSe_2_ (M: Li, Na and K). *Sci. Rep*. **6**, 29292; doi: 10.1038/srep29292 (2016).

## Supplementary Material

Supplementary Information

## Figures and Tables

**Table 1:** List of representative samples prepared in this study.

M	x	*T*_c_	*T*_c_^onset^	*r*_ion_
	(nominal value)	(K)	(K)	(Å)
Na	0.3	5.0	6.0	1.02
Na	0.5	4.8	6.0	1.02
Na	0.5	5.0	6.0	1.02
Na	0.6	4.7	6.0	1.02
Na	0.6	4.7	6.0	1.02
Na	0.8	5.0	6.0	1.02
Na	0.8	5.0	6.0	1.02
Na	1.0	4.7	6.0	1.02
Li	0.5	5.0	6.5	0.76
K	0.5	5.3	7.5	1.38
Sr	0.2	5.0	7.0	1.18
