# Supplementary material for: Emergence of superconductivity in (NH3)yMxMoSe2 (M: Li, Na and K)
Source: Sci Rep. 2016 Jul 11;6:29292. doi: 10.1038/srep29292 (PMC4941414; doi:10.1038/srep29292)
Supplement: Supplementary Information [file srep29292-s1.pdf]

Supplementary Information

Emergence of superconductivity in $(NH_3)_yM_xMoSe_2$ (M: Li, Na and K)

Xiao Miao[1], Saki Nishiyama[1], Lu Zheng[1], Hidenori Goto[1], Ritsuko Eguchi[1], Hiromi Ota[2], Takashi Kambe[3], Kensei Terashima[1], Takayoshi Yokoya[1,4], Huyen T. L. Nguyen[5], Tomoko Kagayama[5], Naohisa Hirao[6], Yasuo Ohishi[6], Hirofumi Ishii[7], Yen-Fa Liao[7] and Yoshihiro Kubozono[1,4*]

[1]Research Laboratory for Surface Science, Okayama University, Okayama 700-8530, Japan

[2]Advanced Science Research Centre, Okayama University, Okayama 700-8530, Japan

[3]Department of Physics, Okayama University, Okayama 700-8530, Japan

[4]Research Centre of New Functional Materials for Energy Production, Storage and Transport, Okayama University, Okayama 700-8530, Japan

[5]Centre for Science and Technology under Extreme Conditions, Osaka University, Osaka 560-8531, Japan

[6]SPring-8 / JASRI, Hyogo 679-5198, Japan

[7]National Synchrotron Radiation Research Center, Hsinchu 30076, Taiwan

*Corresponding author: kubozono@cc.okayama-u.ac.jp

Contents of Supplementary Information

1. Photograph of $MoSe_2$ single crystals is shown in Figure S1a, and X-ray diffraction pattern of a small piece of $(NH_3)_yNa_{0.5}MoSe_2$ is shown in Figure S1b.
2. Temperature dependence of magnetic susceptibility of pure $MoSe_2$ is shown in Figure S2.
3. EDX spectrum of pure $MoSe_2$ is shown in Figure S3.
4. EDX spectrum of pure $(NH_3)_yNa_{0.5}MoSe_2$ is shown in Figure S4.
5. Temperature dependence of magnetic susceptibility of $(NH_3)_yNa_{0.5}MoSe_2$ prepared by Na-doping of polycrystalline $MoSe_2$ is shown in Figure S5.
6. Temperature dependence of magnetic susceptibility of $(NH_3)_yNa_{0.5}MoSe_2$ exhibiting a high Meissner fraction (~25%) is shown in Figure S6.
7. M / H – T plots (ZFC mode) of $(NH_3)_yNa_{0.5}MoSe_2$ at different H's is shown in Figure S7. The H – T_c^{onset} plot is also shown.
8. Positions where electron density was observed in the crystal lattice by the Fourier transform of XRD of a single crystal of $(NH_3)_yNa_{0.5}MoSe_2$ is shown in Figure S8. The yellow circles correspond to the positions of electron density due to a Na atom.
9. Table S1 lists crystallographic data of $MoSe_2$.
10. Table S2 lists crystallographic data of $(NH_3)_yNa_{0.5}MoSe_2$.

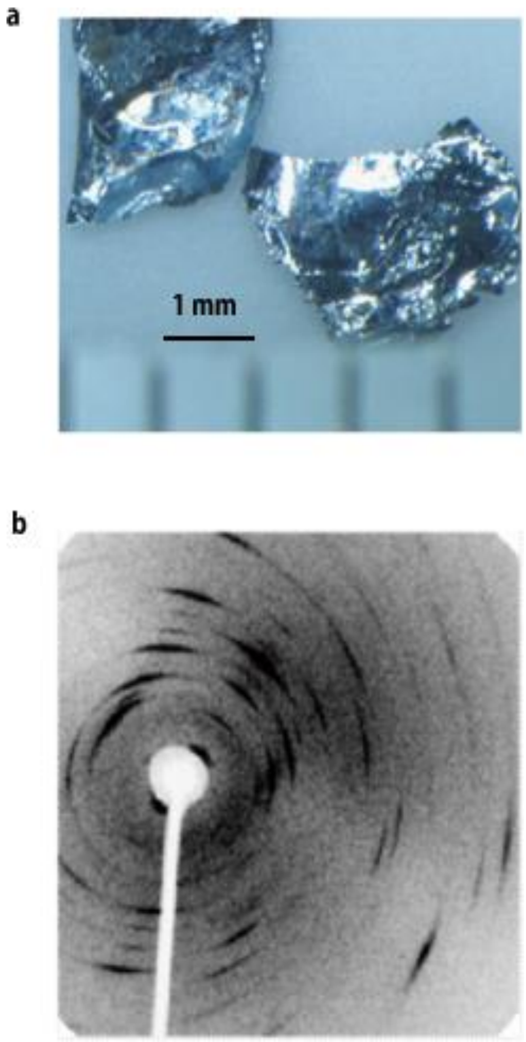

Figure S1. (a) Photograph of agglomerations of $MoSe_2$. A small piece of this agglomeration was single crystal as evidenced from a successful single-crystal X-ray structure analysis. (b) X-ray diffraction of a small piece of $(NH_3)_yNa_{0.5}MoSe_2$, showing the streaky lines.

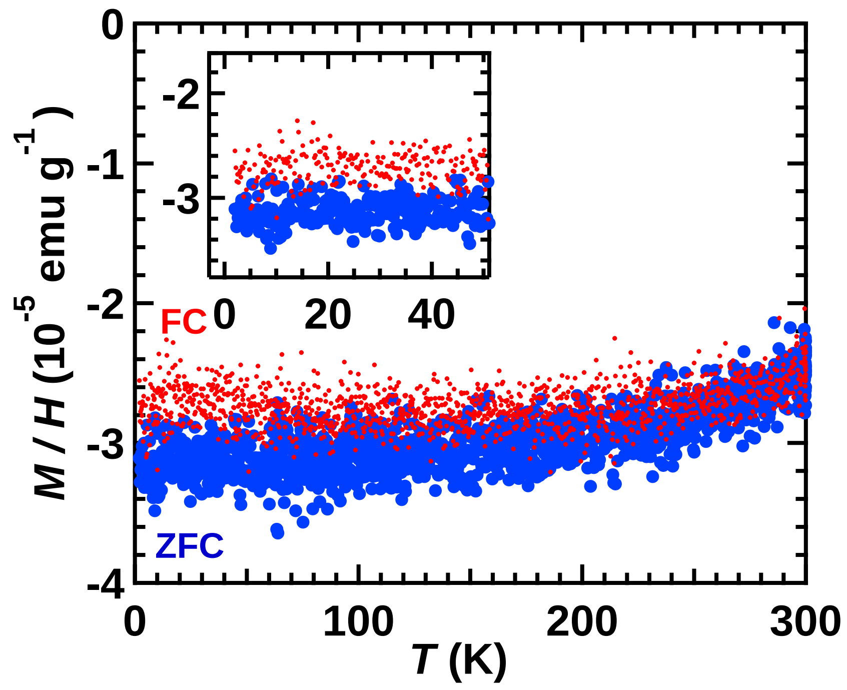

Figure S2. M / H – T plots of $MoSe_2$ agglomerations in ZFC and FC modes.

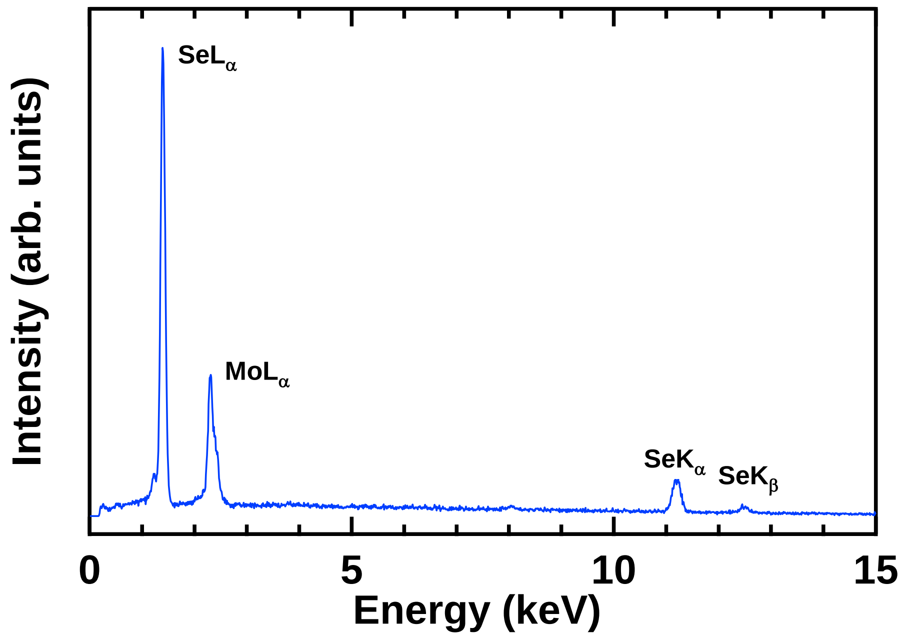

Figure S3. EDX spectrum of pure $MoSe_2$.

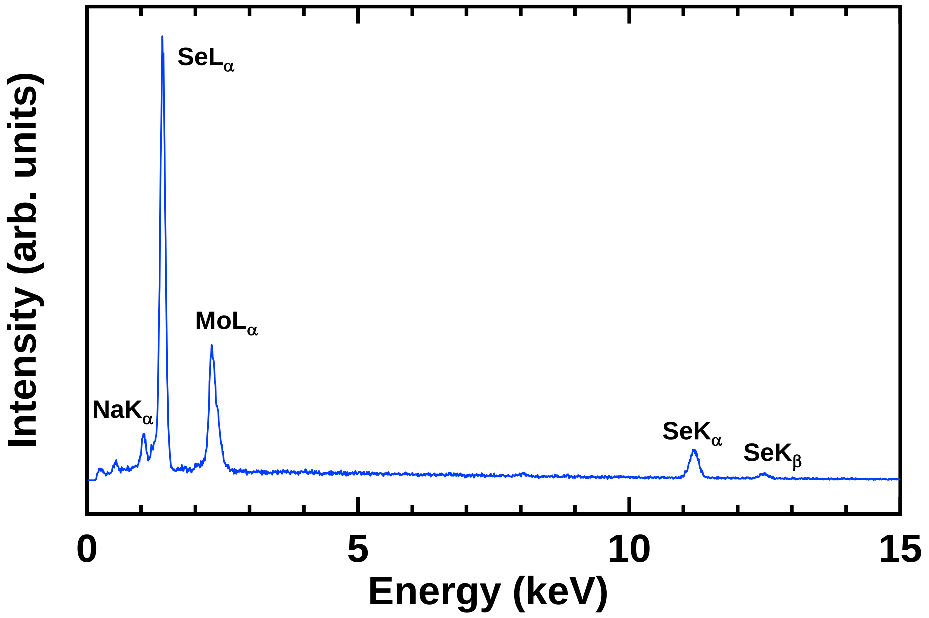

Figure S4. EDX spectrum of $(NH_3)_yNa_{0.5}MoSe_2$.

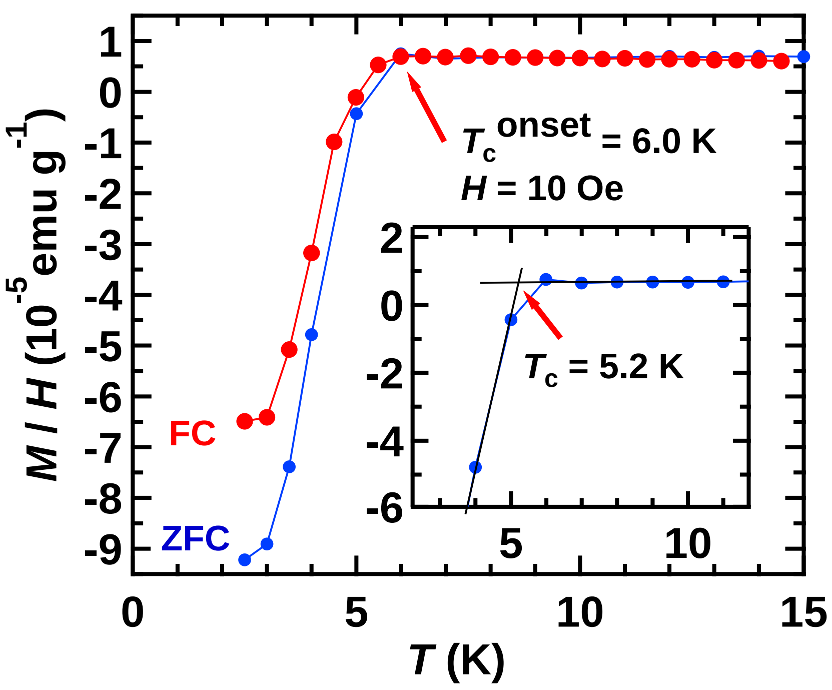

Figure S5. M / H – T plot of $(NH_3)_yNa_{0.5}MoSe_2$ prepared by Na-doping of polycrystalline $MoSe_2$ powder, in ZFC and FC modes.

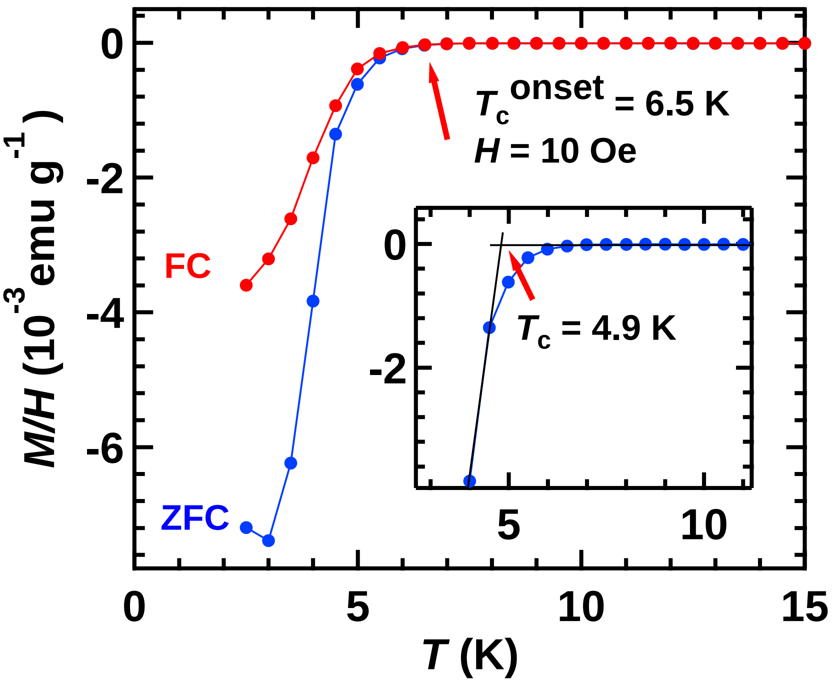

Figure S6. M / H – T plots of $(NH_3)_yNa_{0.5}MoSe_2$ under ZFC and FC modes. The Meissner fraction was approximately 25% at 2.5 K.

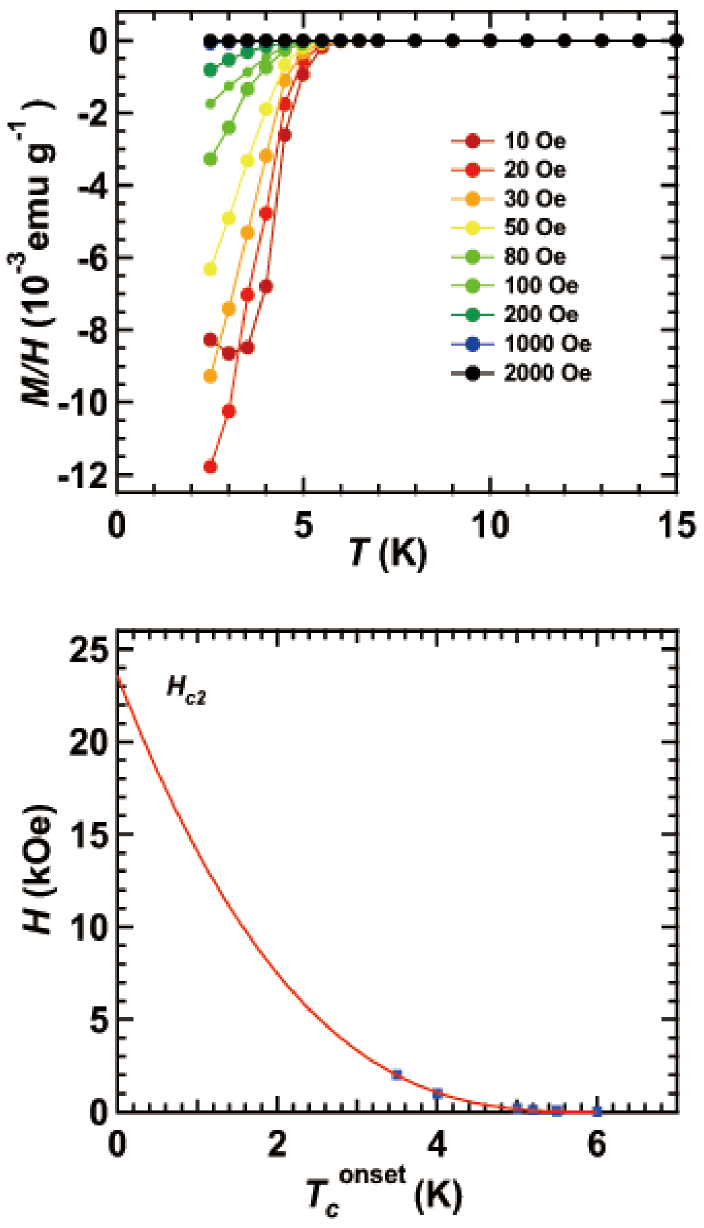

Figure S7. (top) M / H – T plots of $(NH_3)_{0.4(1)}Na_{0.41(1)}MoSe_{2.04(1)}$ at different H's in ZFC mode and (bottom) H – T_c^{onset} plot. The fitting curve was recorded with H_{c2} = $A(T_c - T)^{\alpha}$.

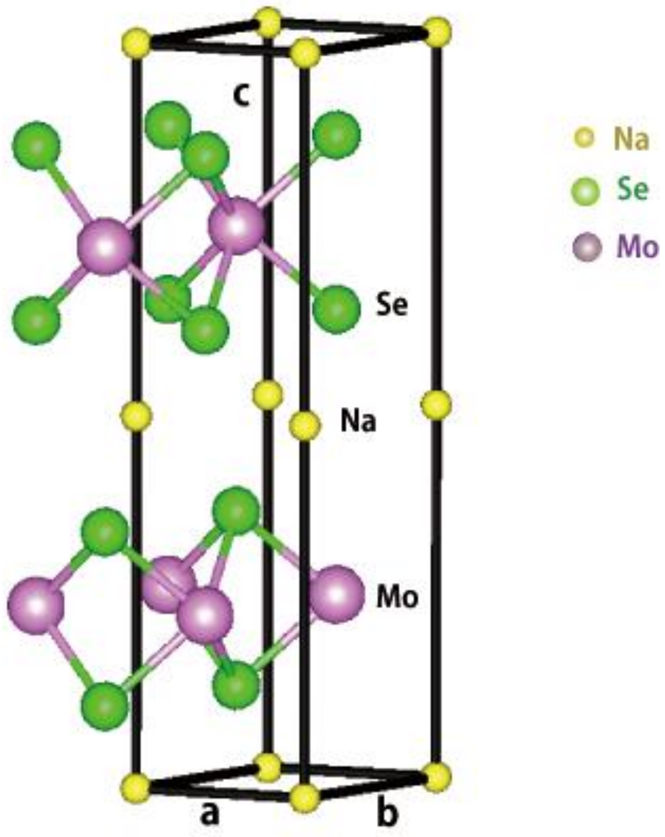

Figure S8. Positions where electron density was observed in the crystal lattice by the Fourier transform of XRD of a single crystal of $(NH_3)_yNa_{0.5}MoSe_2$. The yellow circles correspond to the positions of electron density due to a Na atom.

Table S1. Atomic coordinates of a single crystal of $MoSe_2$

atom	site	*x*	*y*	*z*	B_{eq}	occupancy
Mo	2*c*	0.33333	0.66667	0.25000	0.24(3)	1/12
Se	4*f*	0.33333	0.66667	0.62096(8)	0.26(3)	1/6

Table S2. Atomic coordinates of a single crystal of $(NH_3)_yNa_{0.5}MoSe_2$.The atomic coordinates were obtained from single-crystal XRD analysis. However, the single crystal XRD analysis was not completely refined because of the streak patterns (see text).

atom	site	*x*	*y*	*z*	B_{eq}	occupancy
Mo	2*c*	0.33333	0.66667	0.25000	20(3)	1/12
Se	4*f*	0.33333	0.66667	0.633(8)	25(3)	1/6
Na	2*a*	0.00000	0.00000	0.00000	17(4)	1/12
